# RAB4A GTPase regulates epithelial-to-mesenchymal transition by modulating RAC1 activation

**DOI:** 10.1186/s13058-022-01564-6

**Published:** 2022-10-28

**Authors:** Subbulakshmi Karthikeyan, Patrick J. Casey, Mei Wang

**Affiliations:** 1 Program in Cancer Stem Cell Biology, Duke-NUS Medical School, 8 College Road, Singapore, 169857 Singapore; 2 Department of Pharmacology and Cancer Biology, Duke University Medical Center, Durham, NC 27710 USA; 3 Department of Biochemistry, National University of Singapore, Singapore, 117596 Singapore

**Keywords:** Epithelial–mesenchymal transition (EMT), Cancer cell self-renewal, Stemness, RAB4A, RAC1, Cell invasion, Metastasis

## Abstract

Epithelial-to-mesenchymal transition (EMT) is a critical underpinning process for cancer progression, recurrence and resistance to drug treatment. Identification of new regulators of EMT could lead to the development of effective therapies to improve the outcome of advanced cancers. In the current study we discovered, using a variety of in vitro and in vivo approaches, that RAB4A function is essential for EMT and related manifestation of stemness and invasive properties. Consistently, RAB4A suppression abolished the cancer cells’ self-renewal and tumor forming ability. In terms of downstream signaling, we found that RAB4A regulation of EMT is achieved through its control of activation of the RAC1 GTPase. Introducing activated RAC1 efficiently rescued EMT gene expression, invasion and tumor formation suppressed by RAB4A knockdown in both the in vitro and in vivo cancer models. In summary, this study identifies a RAB4A-RAC1 signaling axis as a key regulatory mechanism for the process of EMT and cancer progression and suggests a potential therapeutic approach to controlling these processes.

**Supplementary Information:**

The online version contains supplementary material available at 10.1186/s13058-022-01564-6.

## Background

Despite recent progress in the understanding of cancer cell signaling and targeted therapy, metastatic disease of solid cancers remains the major cause for cancer-related mortality [1]. A key challenge for the treatment of metastatic cancer is to identify vital regulators that underlie cancer progression [2]. Epithelial–mesenchymal transition (EMT), a highly regulated process, is intricately connected to invasion and metastasis, cancer cell stemness [3], and recurrence in epithelial cancers [3–8]. Since the role of EMT in cancer progression transcends individual cancer types or tissue origin, its modulation can potentially offer a path to treat a broad range of progressive cancers [8]. Understanding the underlying molecular regulation of EMT, therefore, is important not only for deepening our understanding of cancer progression, but also for identifying therapeutic targets.

RAB4A, a member of Ras superfamily of GTPases, is a master regulator of intracellular vesicular trafficking most known for its role in the rapid recycling of membrane proteins to the cell surface [9–11]. Many of these cell surface proteins, such as integrins and growth factor receptors, are known to transmit oncogenic stimuli that lead to cancer formation and progression [12–15]. Indeed, TCGA analysis has revealed that increased Rab4A expression predicts poor overall survival of breast cancer patients [15–18]. A major gap in knowledge is the molecular mechanism of RAB4A involvement in cancer initiation and progression.

RAC1, a key member of the RHO family of small GTPases, has known roles in cancers such as transformation, invasion, angiogenesis and survival [19–24]. RAC1 activity is found to be elevated in many cancer types such as breast, gastric, and oral carcinomas [22]. Although the activation of RAC1 responds to various stimuli, RAB4A has not been previously identified as an upstream regulator for RAC1, particularly in the progression of cancer. This study identifies an involvement of RAB4A, through its regulation of RAC1 activation, in the regulation of EMT, stemness and invasion—all major characteristics of cancer progression, and ultimately patient survival [15, 25–28].

## Materials and methods

### Cell lines and culture conditions

MDA-MB-231, HEK 293T, PC3, MCF7 and SNB19 cells were obtained from American Type Culture Collection (ATCC) (Rockville, MD), tested negative for mycoplasma and cultured according to ATCC protocols. The RAB4A stable knockdown cells and the RAB4A or RAC1 stable overexpression cells were established as described in detail previously [15].

### In vitro assays

Transient transfections were carried out as described previously [29, 30]. RAB4A siRNA sequences are: 5′-CACCGUUAGAUGUGUAUG-3′ and 5′-UUACAUACACAUCUAACG-3′. ITGβ3 shRNA sequences are: GCTCATTGTTGATGCTTAT and GAGGCCACGTCTACCTTCA.

For proliferation assays, cells were seeded in a 96 well plate at low confluency and cultured in the IncuCyte ZOOM incubator (Essen Bioscience, Michigan, USA). The wells were imaged overtime using the build-in IncuCyte ZOOM live-cell microscope for cell number assessment and statistical analysis [31].

The reagents and method for RNA extraction, cDNA extraction and quantitative PCR analysis are as described previously [30]. The primer sequences are listed in Additional file 1: Table S1; the pre-primed PCR plate for EMT is purchased from Bio-Rad (H384).

Immunoblot and image analysis for protein expression is per lab standard protocol [32]. The primary antibodies are listed in Additional file 1: Table S2.

Invasion assays were performed using Transwell plates with 8.0 μm membrane insert (#3422, Corning, USA) as described previously [33–35].

Sphere formation assays were performed as described previously [32]. Briefly, cells were seeded at 400 cells/well in DMEM-F12 containing 0.5% methyl cellulose (Sigma-Aldrich, MO, USA), B-27 and N2 (Gibco, MD.USA) in low-adherent culture plates (#3474, Corning) and were cultured for two weeks. For serial plating, spheres were treated with Accutase cell dissociation reagent, resuspended and seeded as mentioned above. Sphere count was analyzed using Open CFU software.

Soft agar assays were performed as described previously [36]. In brief, a three -layered setup was prepared using noble agar (Sigma-Aldrich, MO, USA), Bottom: 0.5% agar in DMEM; middle: cells suspended in 0.25% agar and DMEM; top: growth medium. The cells were incubated for three weeks with changing of the growth medium (top layer) every week. The colonies were stained using methylthiazolyldiphenyl-tetrazolium bromide (MTT) (Sigma-Aldrich, MO, USA) as per manufacturer’s protocol and visualized by normal light microscopy. Quantification of colonies was performed using Open CFU software.

RAC1-GTP pulldown and analysis were performed using a kit from Cytoskeleton, Inc. (BK035; Denver, CO) according to the manufacturer’s protocol.

Immunohistochemistry or hematoxylin and eosin stain was performed as described previously [37]. Multiple antigens were analyzed (Additional file 1: Table S2) using OPAL-7 manual IHC kit (Perkin Elmer Inc., Cat no: NEL811001KT) kit according to the manufacturer’s protocol. Images were acquired on Leica TCS SP8 confocal microscope at the core facility. Nuclear cytoplasmic ratio was measured using immunofluorescence images taken in chamber slides as described previously [38]. Images were analyzed using ImageJ software.

### Animals and xenografts

All animals were treated in accordance with the IACUC Guidelines (protocol No. 2021/SHS/1627). Briefly, for all the cell lines tested, 0.5 × 10^6^ cells in matrigel were injected into the inguinal mammary fat pad of NOD-SCID-Gamma female mice that were 8–10 weeks old [35, 39]. When the tumors reached 1 cm^3^ (L·X·W^2^/2), the mice were euthanized and tumors were removed and processed for the indicated analyses.

### Statistical analysis

Data are presented as the mean ± standard error of the mean and represent at least three independent biological replicates. Statistical analysis was carried out using GraphPad Prism software (GraphPad, La Jolla, CA). Statistical significance was determined by Student’s unpaired t-test, one-way ANOVA, or two-way ANOVA. ANOVAs were followed with Dunnett’s multiple comparison or Tukey’s post hoc test. *p* < 0.05 considered significant.

## Results

### RAB4A is essential for tumor formation and in vitro sphere formation in the serial replating assay

**Fig. 1:**
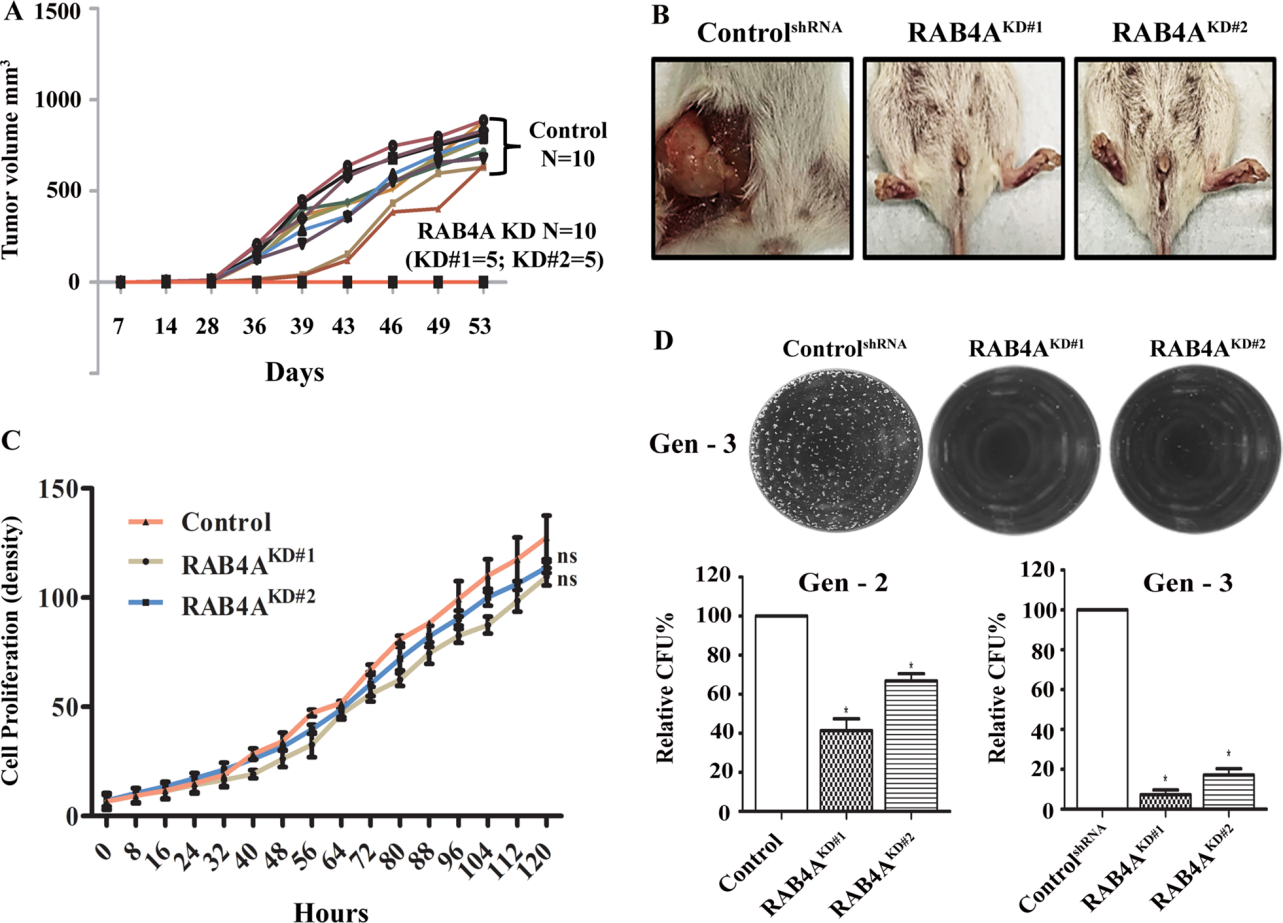
Suppression of RAB4A abolishes tumor formation by inhibiting cancer cell self-renewal/stemness. **A**, **B** Orthotopic mammary fat pad tumor formation study. **A** Shown are the growth rates of tumors derived from MDA-MB-231 breast cancer cells expressing control shRNA (*N* = 10), or shRNA targeting RAB4A KD#1 or RAB4A KD#2 (5 mice in each group, totaling *N* = 10). Since no tumors formed in the KD groups, all ten tumor growth time points fall on the X-axis. **B** Representative in situ tumor images from the study described in (**A**). The cells were implanted in the mammary fat pad in NOD-SCID mice. The in vivo tumor formation study comparing control and RAB4A KD cells was biologically repeated with similar outcome. **C** Cell proliferation under adherent culture conditions of MDA-MB-231 cells expressing control shRNA or two different RAB4A shRNAs as indicated. Proliferation was monitored and recorded continuously for 120 h using IncuCyte live-cell imaging. **D** Serial replating sphere formation assay on the same cell groups as in **C** for self-renewal/stemness analysis. Top: representative microscopic images of the third replating (Gen-3) spheres; bottom: quantification of relative sphere numbers from second replating (Gen-2) and third replating (Gen-3) assays. The data was analyzed using OpenCFU and Prism software. *p < 0.05

While The Cancer Genome Atlas (TCGA) database suggests that RAB4A overexpression in breast cancer patients predicts poor overall survival [15], the functional contribution of RAB4A to tumor development and progression has not been well-evaluated, hence the motivation for this study. To assess this, stable RAB4A knockdown with two different targeting shRNAs (RAB4A KD#1 and RAB4A KD#2) was established in MDA-MB-231 cells. In the orthotopic mammary fat pad model, we found that efficient RAB4A knockdown (Additional file 1: Fig. S1A) completely abolished tumor formation when the cells were implanted in the NOD-SCID mouse mammary fat pad (Fig. 1A and B). Strikingly, the five implants for each of the RAB4A KD#1 and KD#2 cells, totaling ten mice in the RAB4A KD group, formed no tumors, while all ten implants for the control KD cells formed tumors. Interestingly, RAB4A knockdown had no significant growth impact under adherent culturing condition (Fig. 1C), suggesting that the effect of RAB4A on tumor formation is not through the regulation of short-term proliferation.

Serial replating sphere formation assay is commonly used to evaluate the self-renewal or stemness property of cancer cells. By dissociating the cells in the sphere and replating for subsequent round of culturing in the serum-free DMEM with F-12 and N27 supplement, the replicating ability of the non-stem cells is exhausted, leaving only the so-called stem cells to form spheres after replatings. Using this assay, we observed that, although RAB4A suppression had little impact on the first seeding, it caused progressive reduction of sphere formation with each subsequent re-seeding (Fig. 1D). This assay result suggests that RAB4A is essential for the self-renewal ability/stemness of the cancer cells that ensures persistent proliferation. Consistent with this notion, soft agar colony formation assay showed that RAB4A knockdown resulted in ≤ 50% reduction in colonies, i.e. midway between the observations made on adherent culture and the serial replating study (Additional file 1: Fig. S1B).

### RAB4A is essential for the EMT process and cancer invasion

**Fig. 2:**
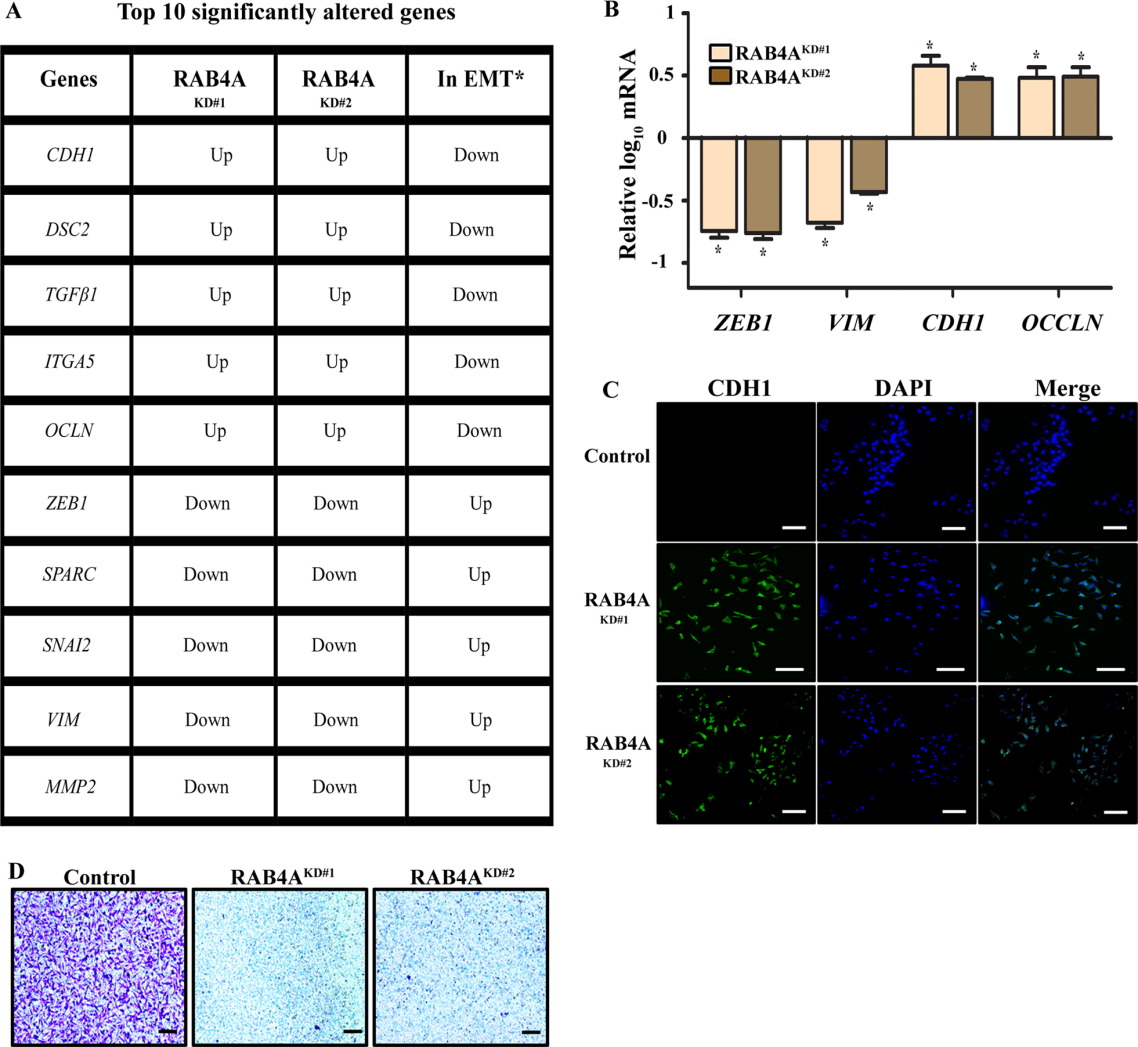
RAB4A knockdown changes the expression of well-known EMT genes in MDA-MB-231 breast cancer cells, in the opposite direction as expected for EMT. The complete profile of the 88 EMT genes whose mRNA levels were determined is presented in Additional file 1: Fig. S2. **A** The table presents the top five up- and down-regulated EMT-related genes in RAB4A knockdown cells in comparison to the control cells; the changes in expression are in the opposite directions as predicted for EMT (last column) [40–42]. **B** qPCR validation of the gene expression of top EMT markers in response to RAB4A knockdown. Data is presented as mean ± SEM (n ≥ 5). “*” represents *p* < 0.05 relative to the expression in control cells, which is set as baseline. **C** Fluorescent microscopic analysis of CDH1 (E-cadherin) expression in MDA-MB-231 cells transfected with control or two different RAB4A KD sequences as in (**A**) and (**B**). DAPI was used to stain the nucleus. Scale bar = 100 µM. **D** Invasion assay of control and RAB4A knockdown MDA-MB-231 cells. Plates were visualized after 24 h in the matrigel invasion culture. Scale bar = 100 µm

Next, we investigated the function of RAB4A in EMT, which is regarded as a critical process in cancer progression and in supporting stemness. Building on the above observations in sphere and tumor formation that linked RAB4A function to cancer cell self-renewal, we evaluated the potential role of RAB4A in EMT. As the EMT program is supported by the transcription of an essential set of genes, we compared the transcription of an established panel of 88 validated EMT-related genes (SAB target list, H384, Bio-Rad) [40–42] in control and RAB4A knockdown cells. We found that the expression of a large fraction of these genes is altered upon the loss of RAB4A (Additional file 1: Fig. S2). Since this panel of genes has experimentally established patterns of up- or down-regulation in the process of EMT from the past studies (3rd column, Additional file 1: Fig. S2) [40–42], we were able to determine whether the RAB4A knockdown-induced expression change of each of the genes is consistent with EMT, or the reversal of EMT. The top five genes that were increased or decreased, respectively, in the RAB4A knockdown cells, but in the reverse directions as expected during EMT process [40–42], are shown in Fig. 2A. Of these, ZEB1, Vimentin, Occludin and E-cadherin (CDH1) are commonly used markers for EMT and cell polarity [40, 42]. Hence, the expression of these genes was individually validated in RAB4A KD cells by quantitative PCR. Indeed, expressions of the EMT-promoting ZEB1 and Vimentin genes were reduced, while those of the epithelial genes CDH1 and OCCLN were elevated, when RAB4A expression was suppressed (Fig. 2B).

E-cadherin (CDH1) is an important epithelial cell marker frequently used in studies of EMT and cell-cell adhesion. CDH1 is consistently down-regulated during EMT, and vice versa for MET—the reverse process. Hence, we performed immunofluorescence analysis of CDH1 on control and RAB4A knockdown MDA-MB-231 cells, which possess strong mesenchymal characteristics. A dramatic elevation of CDH1 protein level was observed when RAB4A is suppressed, providing further support for the role of RAB4A in EMT (Fig. 2C). These gene expression and cell study results are consistent to show that RAB4A regulates the transcription of critical EMT target genes and the EMT process. Functionally, RAB4A knockdown essentially abolished breast cancer cell invasion (Fig. 2D), providing further support for the critical role of RAB4A in supporting EMT.

### RAC1 GTPase is the major downstream effector of RAB4A in the regulation of EMT and cell invasion in epithelial cancers

**Fig. 3:**
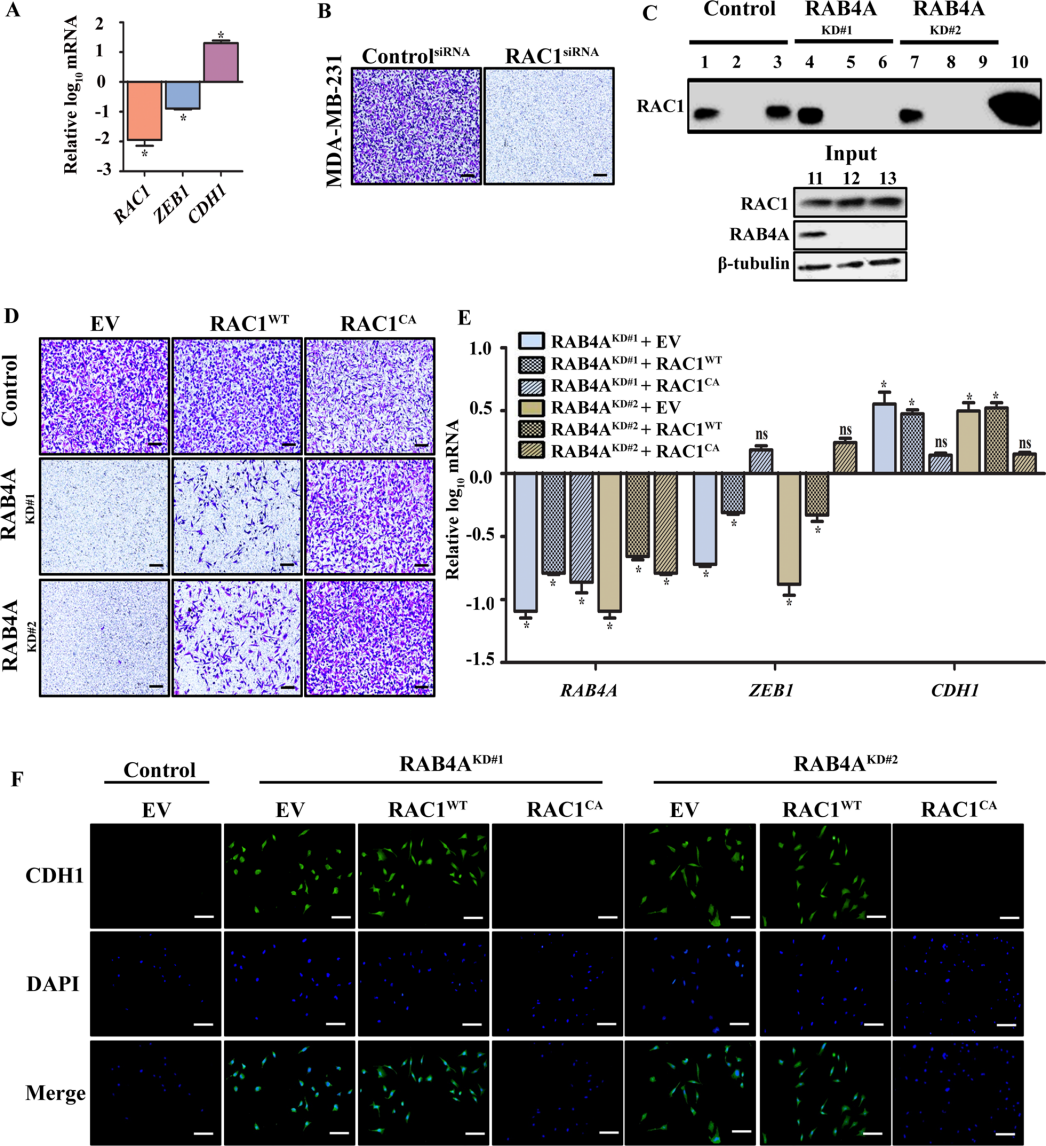
RAB4A regulates RAC1 activation, which is essential for EMT and cell invasion. **A** qPCR analysis of the expression of ZEB1 and CDH1 in response to RAC1 knockdown. “*” represents *p* < 0.05 relative to the expression in control cells, which is set as baseline. **B** Invasion assay assessing the effect of RAC1 knockdown. **C** Pulldown of GTP-bound RAC1 to study the activation status of RAC1 in response to RAB4A knockdown. Lane 1, 4 and 7–lysate incubated with GTPγS; lane 2, 5 and 8–lysate incubated with GDP; lane 3, 6 and 9–lysate only to assess endogenous level of GTP-bound RAC1; lane 10–recombinant RAC1 as immunoblot control. Total lysate input for three cell lysates: lane 11–Control^KD^, lane 12-RAB4A^KD#1^, and lane 13-RAB4A^KD#2^. β-Tubulin is the loading control. **D** Invasion assay to assess the rescue effect of ectopic RAC1 expression in RAB4A knockdown MDA-MB-231 cells. **E** qPCR analysis of RAB4A, ZEB1 and CDH1 expression in the cells in (**D**). Gene expression levels in control shRNA expressing cells set the baseline. **F** Immunofluorescence analysis of CDH1 (E-cadherin) expression in MDA-MB-231 cells with RAB4A KD alone with and without RAC1 expression. DAPI was use to stain the nucleus. Data in the bar graphs of **A** and **E** are presented as mean ± SEM (*n* ≥ 5). “*” and “ns” represent *p* < 0.05 and not significant, respectively, in the comparison to baseline of control cells defined by the x-axis. For invasion study and fluorescence images (**B**, **D** and **E**): scale bar = 100 µM

Our recent study demonstrated that RAB4A regulates integrin β3 localization to the plasma membrane and the outside-in signaling, which is essential for cell migration and invasion [15]. However, the downstream effectors of this function of RAB4A are not adequately defined. In this regard, the RAC1 GTPase has known roles in cytoskeleton organization and cell movement [43, 44]. This connection prompted us to evaluate the relationship between the function of RAC1 and RAB4A. First, we evaluated the impact of RAC1 knockdown in the same cell line—MDA-MB-231; we found that suppression of RAC1 led to the reduction of ZEB1 and elevation of CDH1 gene expression (Fig. 3A) and, phenotypically, completely blocked the cell invasion through matrigel (Fig. 3B).

We next tested whether RAB4A and RAC1 functions are connected in MDA-MB-231 cells. Using the p21-binding-domain (PBD) of PAK that has high affinity only for the active (GTP-bound) form of RAC1, the impact of RAB4A suppression on the activation status of RAC1 was studied by pulldown assays. The level of GTP-bound RAC1 in MDA-MB-231 control cells was comparable to the total RAC1 level as assessed by pulldown following incubation of the lysates with GTPγS (a GTP analogue that irreversibly binds to small GTPases), which suggests that almost all RAC1 in MDA-MB-231 cells exists in the active form (Fig. 3C). Surprisingly, we found that the GTP-bound RAC1 was undetectable in RAB4A knockdown cells (Fig. 3C), supporting a prominent role of RAB4A in the control of RAC1 activation, which has not been previously described.

To evaluate the functional connection between RAB4A and RAC1 in the phenotype of cell invasion, we performed rescue experiments by overexpressing wild-type and a constitutively active form of RAC1 (in this case the Q61L mutant, herein termed RAC1^CA^) in RAB4A knockdown cells. RAC1^WT^ expression resulted in a partial rescue of cell invasion, while RAC1^CA^ expression completely rescued the lost ability of invasion resulted from RAB4A knockdown (Fig. 3D). Since RAB4A regulates key EMT gene expression, qPCR analysis was performed to examine the impact of RAC1 rescue. The baseline expression of each gene was set as that of the control cells—with neither RAB4A knockdown nor exogenous expression of RAC1. As presented earlier, RAB4A knockdown significantly reduced the expression of ZEB1 and increased that of CDH1. RAC1^WT^ expression reversed these effect of RAB4A knockdown, albeit not to the baseline, whereas the expression of RAC1^CA^ completely reversed the gene expression changes to that of the control cells (Fig. 3E). Consistent with the gene expression, immunofluorescence analysis demonstrated that CDH1 expression was undetectable in the highly mesenchymal parental MDA-MB-231 cells. RAB4A knockdown elevated the expression of CDH1 protein, which, upon RAC1^CA^ expression, was suppressed to the undetectable level, reversing the effect of RAB4A knockdown (Fig. 3F). The expression of RAC^WT^ was not sufficient to reverse the inhibitory effect of RAB4A knockdown on CDH1 expression. These results of RAB4A regulation on RAC1 activation, and the ability of activated RAC1 to rescue the effects on cell invasion and EMT gene expression of RAB4A knockdown illustrate that RAC1 is a major mediator of RAB4A-regulation of cancer progression.

### RAC1 serves as a mediator for RAB4A-induced cancer stemness

**Fig. 4:**
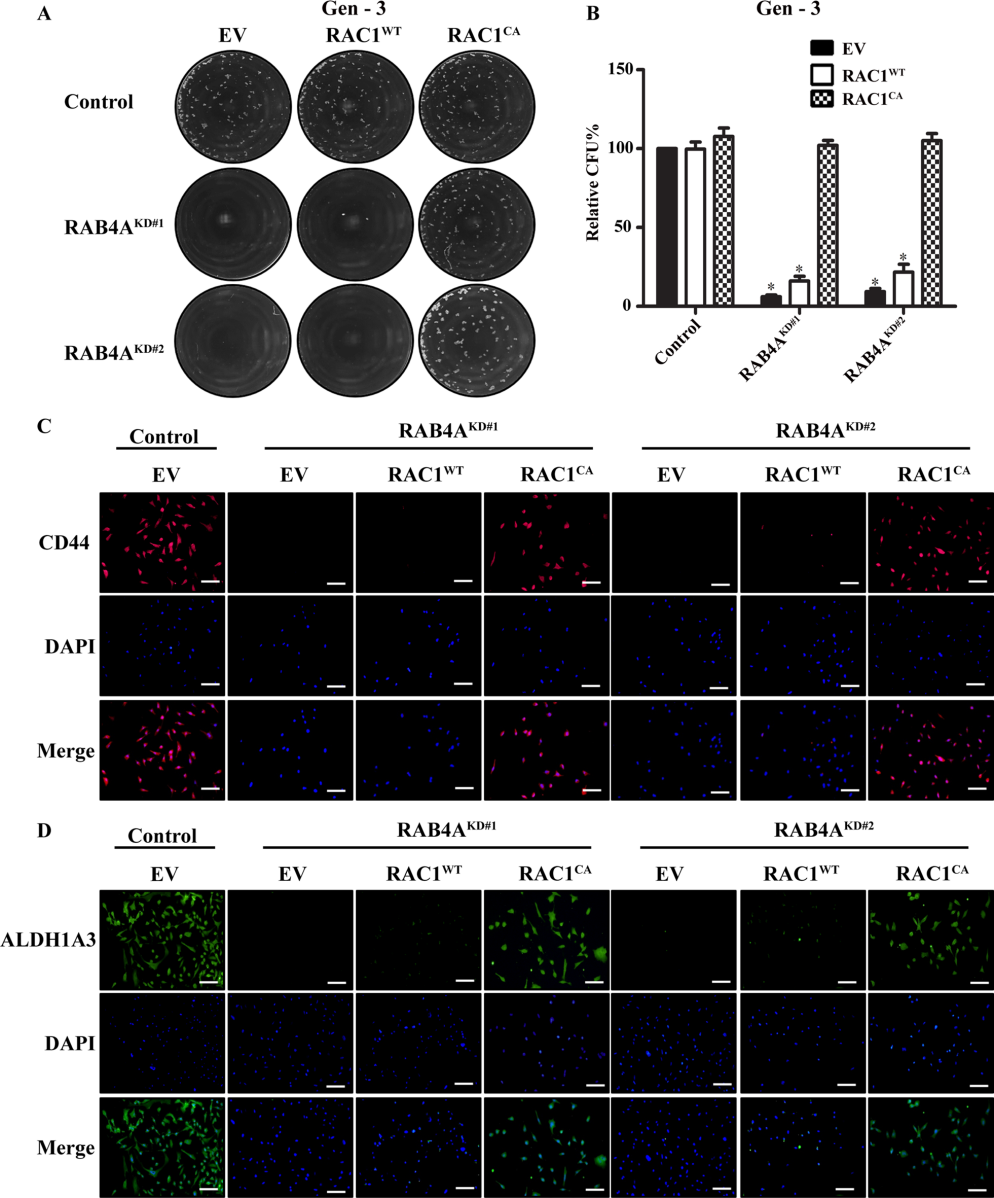
RAC1 is the major downstream effector of RAB4A regulation of cancer stemness. **A**, **B** Serial replating sphere formation assay on MDA-MB-231 cells with RAB4A knockdown alone and in combination with RAC1 overexpression. **A** Representative microscopic images of the third-generation (Gen-3) replating spheres. **B** Quantification of Gen-3 sphere numbers analyzed using OpenCFU and Prism software. *p < 0.05. **C**, **D** Fluorescent microscopic analysis of widely used stemness markers of breast cancer cells (CD44, ALDH1A3) on the same cell groups as in (**A**). Representative images are shown for CD44 staining **C** and ALDH1A3 staining **D**. DAPI was used to stain the nucleus. Scale bar = 100 µM

As changes in cancer stemness are associated with the process of EMT, we next assessed the effect of RAC1 rescue in terms of the self-renewal/long term proliferation potential in RAB4A knockdown cells as assessed by serial replating sphere formation assay. Consistent with our earlier results, RAB4A knockdown nearly abolished the sphere formation at the 3rd replating (Gen-3). As hypothesized from its role in EMT, expressing constitutively active RAC1^CA^ restored sphere formation ability, while RAC1^WT^ yielded no significant rescuing effect (Fig. 4A and B). We also evaluated the expression of ALDH1A3 and CD44, markers for breast cancer stem cells, in both control and RAB4A knockdown cells, and in the RAB4A knockdown cells that concurrently express either RAC1^WT^ or RAC1^CA^. Consistent with the long term sphere formation result, RAB4A knockdown markedly reduced the expression of stem cell markers ALDH1A3 and CD44, which was rescued by the expression of RAC1^CA^, but not RAC1^WT^ (Fig. 4C and D). Additionally, we tested the effect of RAC1 rescue of RAB4A knockdown on cell proliferation under both adherent and anchorage-independent culture conditions. Consistent with the observation made with RAB4A knockdown alone, expression of neither RAC1^WT^ nor RAC1^CA^ significantly impacted 2D proliferation (Additional file 1: Fig. S3A), while RAC1^CA^ but not RAC1^WT^, partially rescued the soft agar colony formation (Additional file 1: Fig. S3B). The discrepancy between results from the serial replating sphere formation, adherent culture and the soft agar growth assays support the role of RAB4A and RAC1 in stemness/self-renewal rather than short-term proliferation.

Based on our recent discovery of RAB4A regulation of integrin β3 (ITGβ3) recycling [15], we evaluated the role of ITGβ3 in the RAB4A to RAC1 signaling. To this end, we studied the effect of ITGβ3 overexpression on MCF7 breast cancer cells, which have the characteristics of low RAB4A and ITGβ3 expression, low invasiveness, and predominantly epithelial phenotype. The expression of ITGβ3 markedly stimulated cell invasion (Additional file 1: Fig. S4A), and, interestingly, stimulated RAC1 activation in MCF7 cells that have low endogenous RAC1 activity (Additional file 1: Fig. S4B). ITGβ3 expression also led to a marked induction of ZEB1 and suppression of CDH1 transcription (Additional file 1: Fig. S4C), consistent with its role in mediating the signaling from RAB4A to RAC1.

In addition to the overexpression studies in the ITGβ3-low MCF7 cells, we also evaluated the outcomes of knocking down ITGβ3 in the ITGβ-high MDA-MB-231 cells. Opposite to overexpression findings noted above, lowering ITGβ3 levels blocked cell invasion (Additional file 1: Fig. S4D), inhibited RAC1 activity (Additional file 1: Fig. S4E), and reduced ZEB1 while induced CDH1 transcription in MDA-MB-231 cells (Additional file 1: Fig. S4F). Taken together, these results established that RAC1 activation is downstream of RAB4A and integrin β3 in the regulation of EMT and cell invasion.

### RAB4A-RAC1 signaling regulates EMT gene expression, cell invasion and cancer stemness in multiple cancer cell lines

**Fig. 5:**
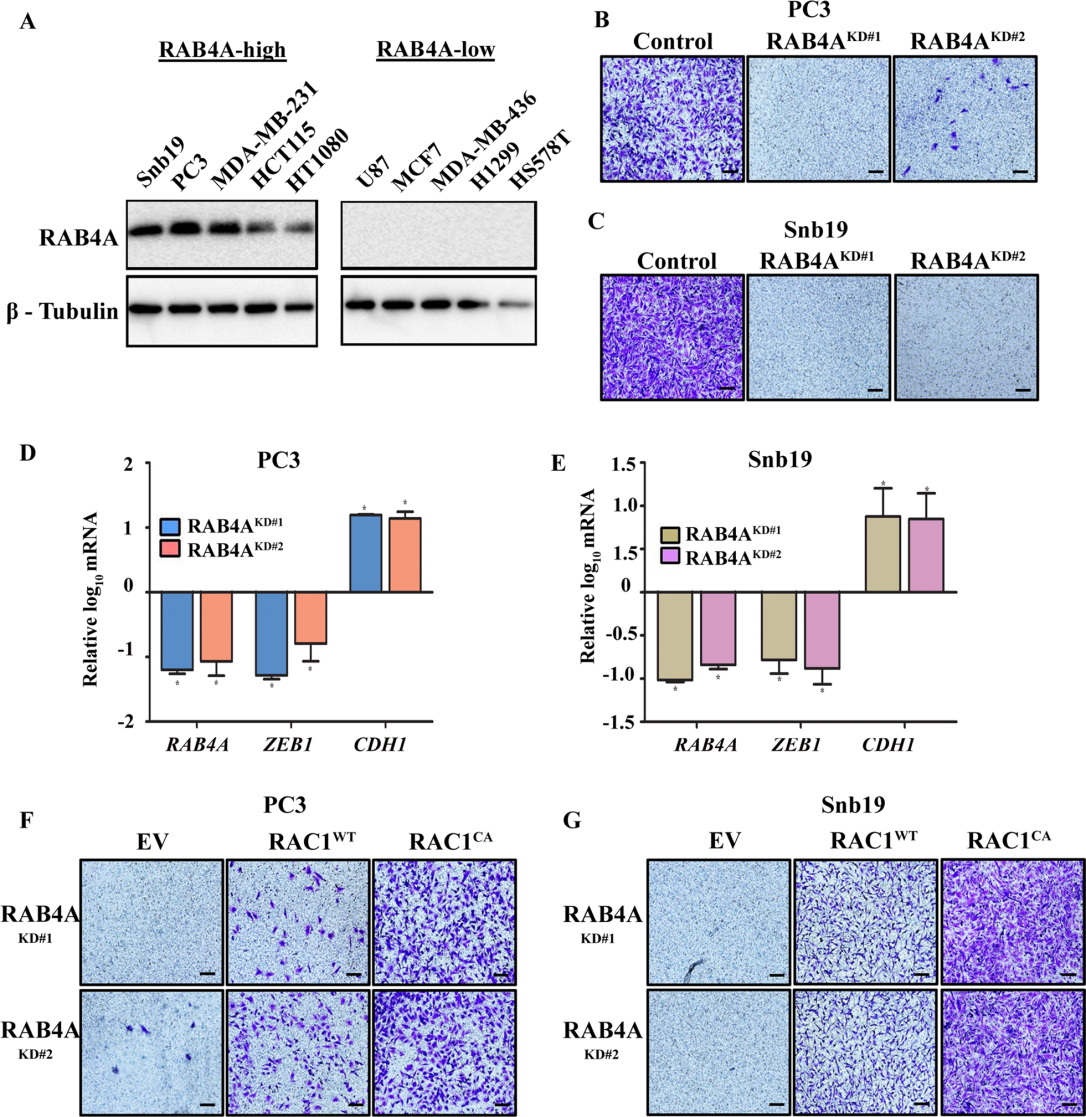
Impact of RAB4A and RAC1 co-manipulation on mesenchymal-epithelial properties and cell invasion in multiple cancer cell lines, **A** Immunoblot analysis of RAB4A protein expression in human cancer cell lines of diverse tissue origins. **B**, **C** Invasion study of the RAB4A high-expressing PC3 **B** and SNB19 **C** cells, with and without RAB4A knockdown. Scale bar = 100 µM. **D**, **E** qPCR analysis to quantify the expression levels of mesenchymal marker ZEB1 and epithelial marker E-Cadherin (CDH1) upon RAB4A knockdown using the groups of cells as in **B**, **C**. The expression levels and changes of these markers are compared to those in the control siRNA cells, which set the baseline. “*”, *p* < 0.05 represents significance compared to control cells. **F**, **G** Invasion study of PC3 **F** and SNB19 **G** cells with RAB4A knockdown with and without concurrent expression of either wild-type RAC1 (RAC1^WT^) or constitutively active RAC1 (RAC1^CA^). Scale bar = 100 µM

The studies described above were all performed in MDA-MB-231 and MCF7 breast cancer cells. To evaluate the broader applicability of the newly-discovered relationship between RAB4A and RAC1 in regulating EMT and cell invasion, we expanded the study to other cancer cell lines. First, we analyzed RAB4A protein levels in a panel of human cancer cell lines of various tissue origins, which identified RAB4A-high and -low groups (Fig. 5A). We then subjected the RAB4A-high PC3 prostate cancer and SNB19 glioblastoma cancer cells to stable RAB4A knockdown, which led to the observation that loss of RAB4A abolished cell invasion in both cell types (Fig. 5B and C). We then evaluated the effect of RAB4A knockdown on EMT gene expression and found that, similar to the observations made on MDA-MB-231 cells, RAB4A silencing decreased ZEB1 and increased CDH1 mRNA levels in both cell lines (Fig. 5D and E). Further, we assessed the rescue effect of expression of RAC1^WT^ and RAC1^CA^ on cell invasion in RAB4A knockdown PC3 and SNB19 cells. We observed that RAC1^CA^ effectively restored the invasiveness of both cell lines, reversing the effect of RAB4A knockdown, while the RAC1^WT^ had much diminished rescue effect (Fig. 5F and G).

**Fig. 6:**
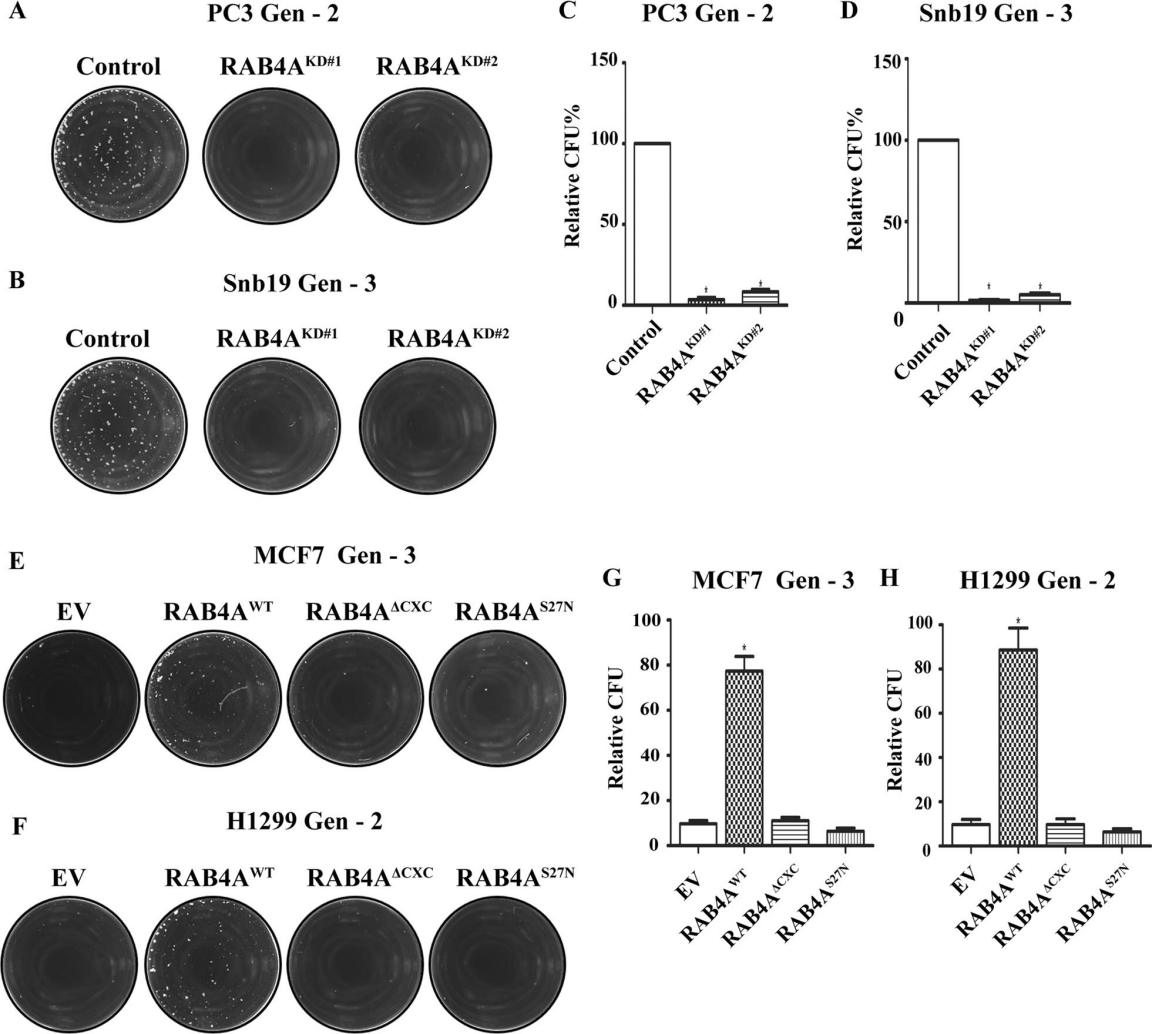
RAB4A is essential for self-renewal in multiple human cancer cell lines. (A-D) Serial replating sphere formation assay on RAB4A-high cells with and without stable RAB4A knockdown. **A**, **B** Representative microscopic images of the replating spheres of PC3 **A** and SNB19 **B** cells. **C**, **D** Quantification of sphere numbers from **A**, **B** analyzed using OpenCFU and Prism software. **E**–**H** Serial replating sphere formation assay on RAB4A-low cells with and without multiple forms of RAB4A stable overexpression. **E**, **F** Representative microscopic images of the replating spheres of MCF7 **E** and H1299 **F** cells. **G**, **H** Quantification of sphere numbers from **E**, **F** analyzed using OpenCFU and Prism software. Data presented in the bar graphs are mean ± SEM (*n* ≥ 5). “*” represents *p* < 0.05 of the comparison made between control and the corresponding group

We then assessed the contribution of RAB4A in proliferation and stemness/self-renewal in PC3 and SNB19 cells. As observed in MDA-MB-231 cells, RAB4A knockdown abolished the third-generation sphere formation (Fig. 6A–D), while it did not alter the 2D cell proliferation (Additional file 1: Fig. S5A and B). Also similar to that observed in MDA-MB-231 cells, suppression of RAB4A in PC3 and SNB19 cells significantly reduced the soft agar colony formation, but the effect was more moderate than the impact on late passage sphere formation (Additional file 1: Fig. S5C and D).

Thus far, our evidence demonstrates the effect of suppressing RAB4A on inhibiting EMT, cell invasion and stemness in RAB4A high-expressing cells such as MDA-MB-231, PC3 and SNB19 cells. To further this correlation, we overexpressed RAB4A in two RAB4A-low cells. The comparison was made between the cells stably expressing wild-type RAB4A (RAB4A^WT^), the C-terminal prenylation modification site deletion mutant (RAB4A^ΔCXC^), and the inactive RAB4A mutant (RAB4A^S27N^). The hypothesis was that, if RAB4A is an essential mediator of stemness in these RAB4A-low cells as in the case of RAB4A-high cells, overexpression of the wild-type RAB4A should facilitate the growth of replating spheres and anchorage-independent colony formation. For this study, we used MCF7 breast cancer and H1299 non-small cell lung cancer cells that have low intrinsic RAB4A expression and low baseline replating sphere formation ability (Fig. 6E–H). We found that the replating sphere formation was significantly increased over that of the parental cells only upon expression of RAB4A^WT^, but neither RAB4A^ΔCXC^ nor RAB4A^S27N^ mutant proteins (Fig. 6E–H). Consistent with the findings in knockdown studies on RAB4A-high cells and the notion that RAB4A impact is on the self-renewal/stemness, we observed no significant changes in adherent culture proliferation (Additional file 1: Fig. S6A and B) and only moderate increase in soft agar colony growth (Additional file 1: Fig. S6C and D). These findings confirm that the regulatory role of RAB4A-RAC1 signaling is not limited to MDA-MB-231 but broadly applicable to many cancer cells—that express RAB4A at high and low levels.

### RAC1 mediates the RAB4A regulation of tumorigenesis in an orthotopic mouse model

**Fig. 7:**
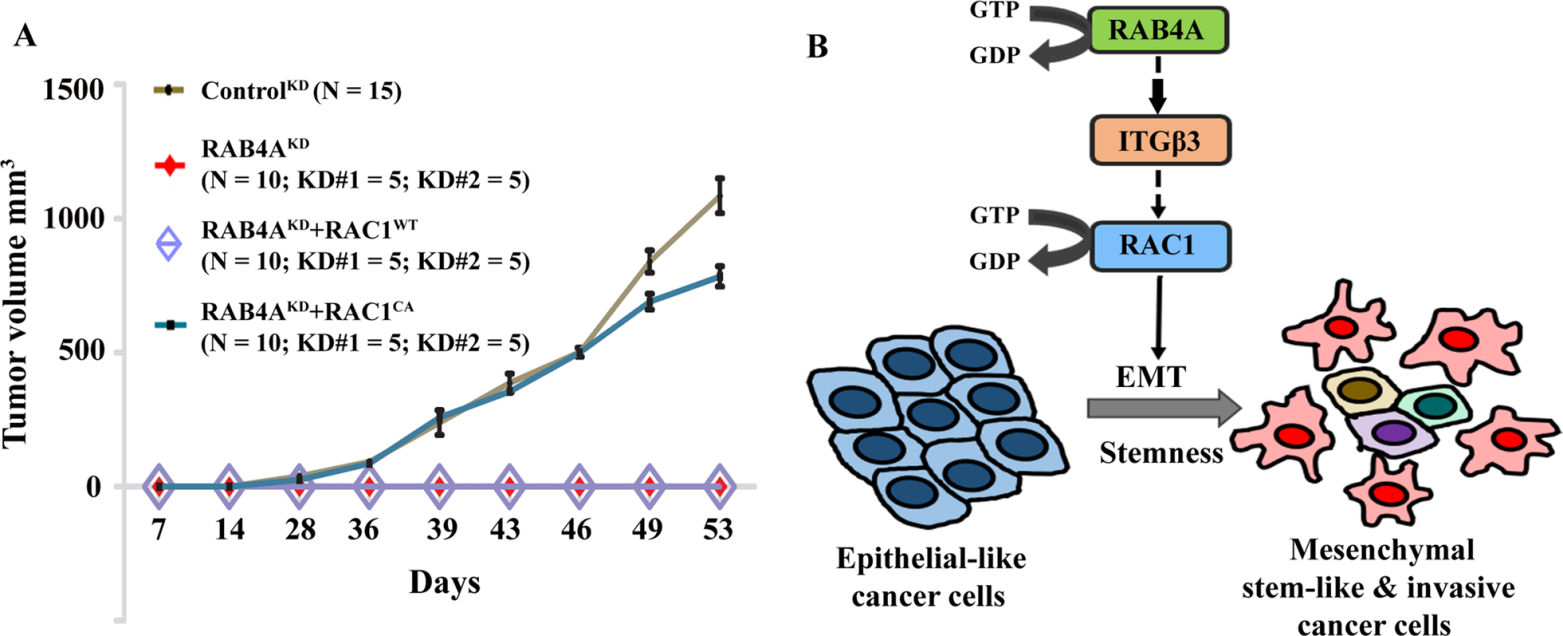
RAC1 restores the MDA-MB-231 cell tumor forming ability that is lost from RAB4A knockdown in the orthotopic mammary fat pad mice model. **A** The formation and growth of tumors derived from control MDA-MB-231 cells, and those with stable RAB4A knockdown alone or concurrent RAB4A knockdown and RAC1 overexpression. The analysis was performed using Prism software. The numbers of mice in each experimental group are: RAB4A KD, 10 mice (RAB4A KD#1 and RAB4A KD#2 includes 5 mice each); combined RAB4A KD with concurrent expression of wild-type RAC1, 10 mice (RAB4A KD#1 + RAC1^WT^ and RAB4A KD#2 + RAC1^WT^ includes 5 mice each); combined RAB4A KD with concurrent expression of constitutive active RAC1, 10 mice (RAB4A KD#1 + RAC1^CA^ and RAB4A KD#2 + RAC1^CA^ includes 5 mice each); control shRNA group, 15 mice. The growth curves of the ten RAB4A KD and ten RAB4A KD + RAC1^WT^ all fall onto the X-axis; the solid red diamond and open purple diamond represent these two groups, respectively. **B** Graphic illustration of the proposed model based on the study results. RAB4A-RAC1 signaling is an essential regulatory pathway of EMT, invasion and stemness, which are critical features of cancer progression. RAB4A acts through integrin β3 to control the activation of RAC1

Taking the RAC1 story further to support its new role as a downstream regulator of RAB4A in EMT, invasion and sphere formation in in vitro settings, we performed an orthotopic in vivo tumor formation study. Specifically, we compared the tumor formation ability of cells derived from MDA‐MB‐231 that expressing either control shRNA or RAB4A shRNA alone or concurrent expression of RAB4A shRNA and either RAC1^WT^ or RAC1^CA^. These cells were implanted into the inguinal mammary fat pad of NOD‐SCID mice. Consistent with our initial observation, RAB4A knockdown cells never form tumors (Fig. 7A, Additional file 1: Fig. S7). However, introducing the expression of RAC1^CA^, but not RAC1^WT^, restored the tumor formation ability of the RAB4A knockdown cells to the level of control cells (Fig. 7A, Additional file 1: Fig. S7), which is consistent with the notion that RAC1 is the primary mediator of RAB4A regulation of tumor formation and progression. The inability of RAC1^WT^ to restore the tumor formation ability is consistent with the conclusion that RAB4A plays a major role in the activation of RAC1; therefore, only the activated RAC1 can perform the function without the help of RAB4A.

In summary of all the evidence, we illustrate in the in vitro and in vivo settings the essential roles of RAB4A in EMT, cell invasion, stemness and tumor formation. Mechanistically, this study identifies RAB4A as an upstream regulator of RAC1 activation, through which RAB4A performs these regulatory functions in cancer signaling and major cancer phenotypes (Fig. 7B).

## Discussion

Despite recent advancements in cancer treatment, the outcome for advanced solid cancers with metastasis remains dismal, and more than 90% of cancer-related deaths being due to metastatic disease [45]. Carcinomas that originate from epithelial tissues become locally invasive and gain the ability to metastasize to distant sites, usually after going through a process of trans-differentiation termed EMT [46]. Given the many cancer cell properties associated with EMT, such as stemness and invasion, targeting EMT regulatory pathways is considered an important strategy for the treatment of solid cancers [46]. To this end, the identification of targetable EMT regulators and pathways is especially important.

In this study, we evaluated the crucial involvement of RAB4A in the regulation of EMT through multiple complementary in vitro and in vivo approaches. While both RAB4A and RAC1 have important cellular functions [12, 22, 23], a RAB4A-to-RAC1 signaling in regulating EMT and supporting stemness features has not been described. We present evidence that RAB4A is a critical regulator of EMT that is the foundation of invasive behavior in many types of human cancer cells. RAB4A loss-of-function alone abolished tumor formation in an orthotopic mammary fat pad mice model and reduced sphere formation in serial seeding of cells, indicating the critical involvement of RAB4A in regulating cancer stemness phenotypes that are often associated with EMT. We further provided evidence to establish an essential role of integrin β3 in mediating the RAB4A regulation of RAC1 activation. The chain of signaling, which supports the major phenotypes of aggressive solid cancers such as EMT, stemness and invasiveness, represents a mechanistic advancement in understanding cancer progression. Considering the unmet need for effective therapeutics for advanced solid cancers, the discoveries described in this work is of translational value.

## Supplementary Information

**Additional file 1**. Supplementary Tables and Figures

## Data Availability

The datasets used and/or analyzed during the current study are available from the corresponding author.
