# Supplementary material for: RAB4A GTPase regulates epithelial-to-mesenchymal transition by modulating RAC1 activation
Source: Breast Cancer Res. 2022 Oct 28;24:72. doi: 10.1186/s13058-022-01564-6 (PMC9615386; doi:10.1186/s13058-022-01564-6)
Supplement: Supplementary file 1 — Additional file 1. Supplementary Tables and Figures [file 13058_2022_1564_MOESM1_ESM.pdf]

I. Supplementary Tables

Supplementary Table 1. List of primers used in the study

Primer	Forward sequence 5'-3'	Reverse sequence 5'-3'
RAB4A	AATCAGGTGAGCTGGACCCAGAAA	ACCTGTGTGCTCTCCTAACAACCA
ZEB1	AGTGGTCATGAAAATGGAAC	AGGTGTAACTGCACAGGGAGC
CDH1	GTCCTGGGCAGAGTGAATTT	GACCAAGAAATGGATGTGTGG
VIM	CGAGGAGAGCAGGATTTCTC	GGTATCAACCAGAGGGAGTGA
OCCLN	AAAGTCCACCTCCTTACAGGC	GGCTGAGAGAGCATTGGTCG
18S	AAGTTCGACCGTCTTCTCAGC	GTTGATTAAGTCCCTGCCCTTTG

Supplementary Table 2. List of antibodies used in immunoblotting, immunohistochemistry and immunofluorescence analysis

Antibody	Cat no	Provider
E-cadherin (CDH1)	3195	Cell signaling
Aldh1A3	GTX110784	Genetex
CD44	GTX102111	Genetex
ITGβ3	ab190147	Abcam
RAB4A	WH0005867M1	Sigma
β-Tubulin	2128	Cell signaling

II. Supplementary Figures

Figure S1. RAB4A knockdown reduces the anchorage independent colony formation.

(A) Immunoblot assessment of RAB4A knockdown efficiency by two targeting RNAi.

(B) Soft agar colony formation of MDA-MB-231 cells with stable RAB4A knockdown using two distinct RNAi sequences ($RAB4A^{KD\#1}$ or $RAB4A^{KD\#2}$) compared to control cells. Top: Representative microscopic images of the colonies formed after 20 days of culture in noble agar; bottom: Relative quantification of colony numbers compared to the control.

Figure S1

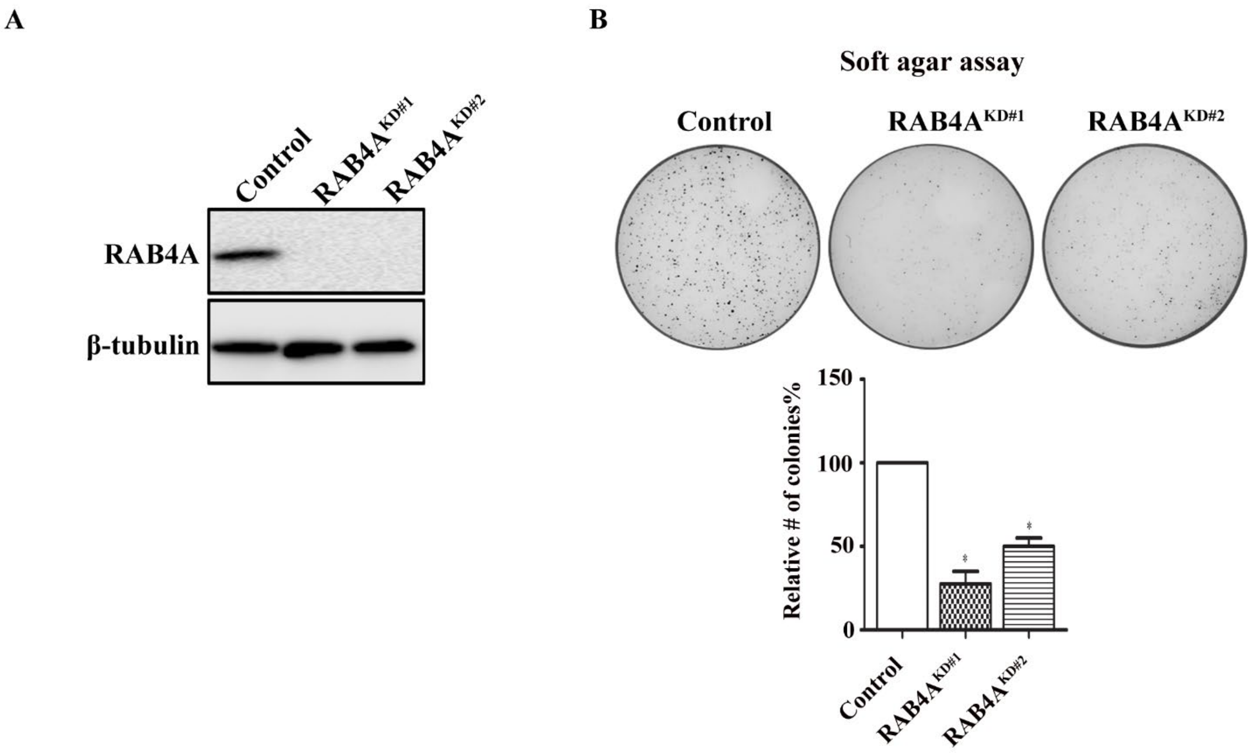

Figure S2. RAB4A regulates EMT gene expression and cell invasion.

Gene expression changes in response to RAB4A silencing using the EMT qPCR multiplex array (SAB target list, H384, Bio-Rad). The first two columns are the comparisons made between the control and RAB4A knockdown cells; the color code indicates increased (red) or decreased (green) expression associated with RAB4A knockdown. The third column labeled “In EMT*” denotes the regulation of expression in a typical EMT process based on published research; red and green indicate genes whose expression is typically up- or down-regulated when cells go through EMT [1-3].

Figure S2

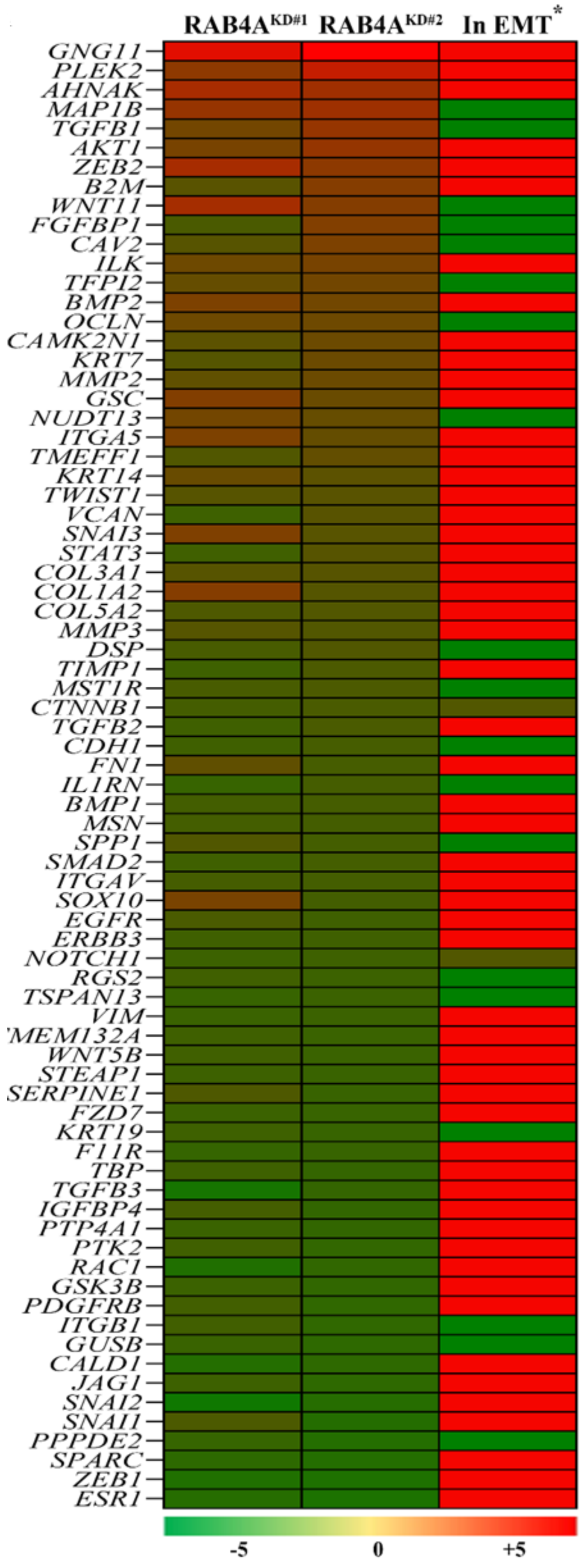
RAB4A KD#1
RAB4A KD#2
In EMT*
GNG11
PLEK2
AHNAK
MAP1B
TGFB1
AKT1
ZEB2
B2M
WNT11
FGFBP1
CAV2
ILK
TFPI2
BMP2
OCLN
CAMK2N1
KRT7
MMP2
GSC
NUDT13
ITGA5
TMEFF1
KRT14
TWIST1
VCAN
SNAI3
STAT3
COL3A1
COL1A2
COL5A2
MMP3
DSP
TIMP1
MST1R
CTNNB1
TGFB2
CDH1
FN1
IL1RN
BMP1
MSN
SPP1
SMAD2
ITGAV
SOX10
EGFR
ERBB3
NOTCH1
RGS2
TSPAN13
VIM
MEM132A
WNT5B
STEAP1
SERPINE1
FZD7
KRT19
F11R
TBP
TGFB3
IGFBP4
PTP4A1
PTK2
RAC1
GSK3B
PDGFRB
ITGB1
GUSB
CALD1
JAG1
SNAI2
SNAI1
PPPDE2
SPARC
ZEB1
ESR1
-5
0
+5

Figure S3. Constitutively active RAC1 expression restores MDA-MB-231 cell colony formation ability that was lost upon RAB4A knockdown, while exerting no significant adherent cell proliferation.

(A) Cell proliferation assay performed using adherent-cultured MDA-MB-231 cells expressing RAB4A shRNA alone or in combination with RAC1^{WT} or RAC1^{CA} overexpression. Proliferation was recorded continuously for 120 hours using IncuCyte live-cell imaging.

(B) Soft agar assay on the same group of cells as in (A). Plates were visualized after 21 days in culture.

Figure S3

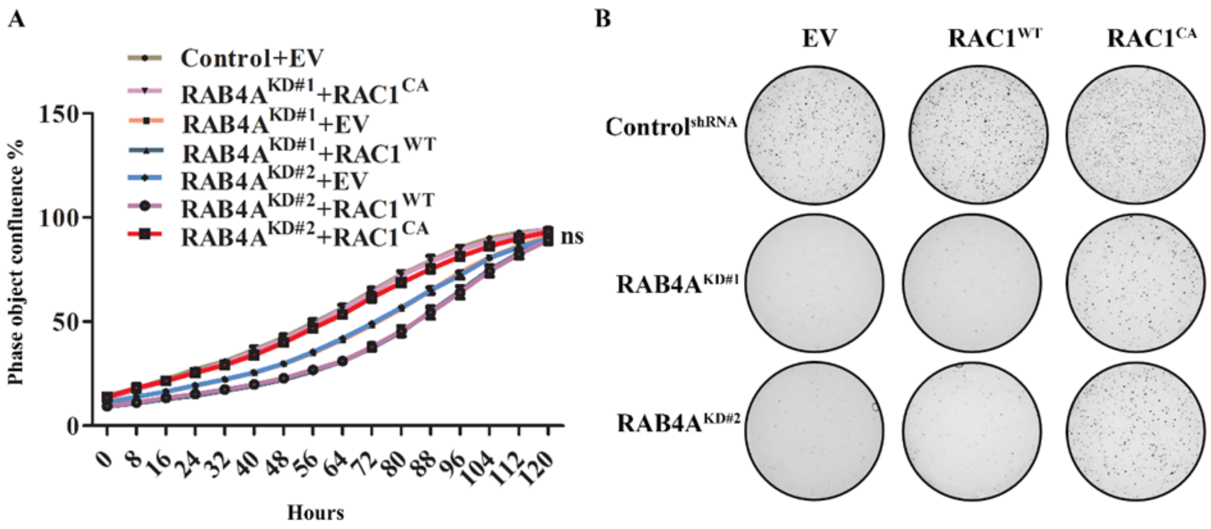

Figure S4. **RAB4A activation of RAC1 involves integrin β3 function.**

(A) Invasion ability of MCF7 breast cancer cells with and without overexpression of integrin β3. Scale bar - 100 µm.

(B) Pulldown quantification of GTP-bound RAC1 from MCF7 breast cancer cells with and without overexpression of integrin β3. Lane 1, 4 – lysate plus GTPγS; Lane 2, 5 – lysate plus GDP; Lane 3, 6 –lysate only; Lane 7 – recombinant RAC1 as immunoblot control. Lane 8, 9 are the total lysate input for MCF7 control cells (EV) and MCF7 expressing integrin β3.

(C) Q-PCR analysis of ZEB1 and CDH1 transcription levels in the cell groups of (A); the baseline was set by mock expression cells (EV). "*": $p < 0.05$ compared to the baseline.

(D) Invasion ability of MDA-MB-231 breast cancer cells with and without integrin β3 knockdown; two different shRNAs were used to establish stable integrin β3 knockdown cells. Scale bar - 100 µm.

(E) Quantification of GTP-bound RAC1 from the cell groups in (D). Lane 1, 4, 7 – lysate plus GTPγS; Lane 2, 5, 8 – lysate plus GDP; Lane 3, 6, 9 – lysate only; Lane 10 – Recombinant RAC1 as SDS PAGE immunoblot control. Total lysate inputs: lane 11 – control shRNA, lane 12 – ITGβ3 shRNA1, lane 13 – ITGβ3 shRNA2.

(F) Q-PCR analysis of ZEB1 and CDH1 transcription levels in the cell groups in (D); the baseline was set by cells that express control shRNA. "*": $p < 0.05$ compared to the baseline.

Figure S4

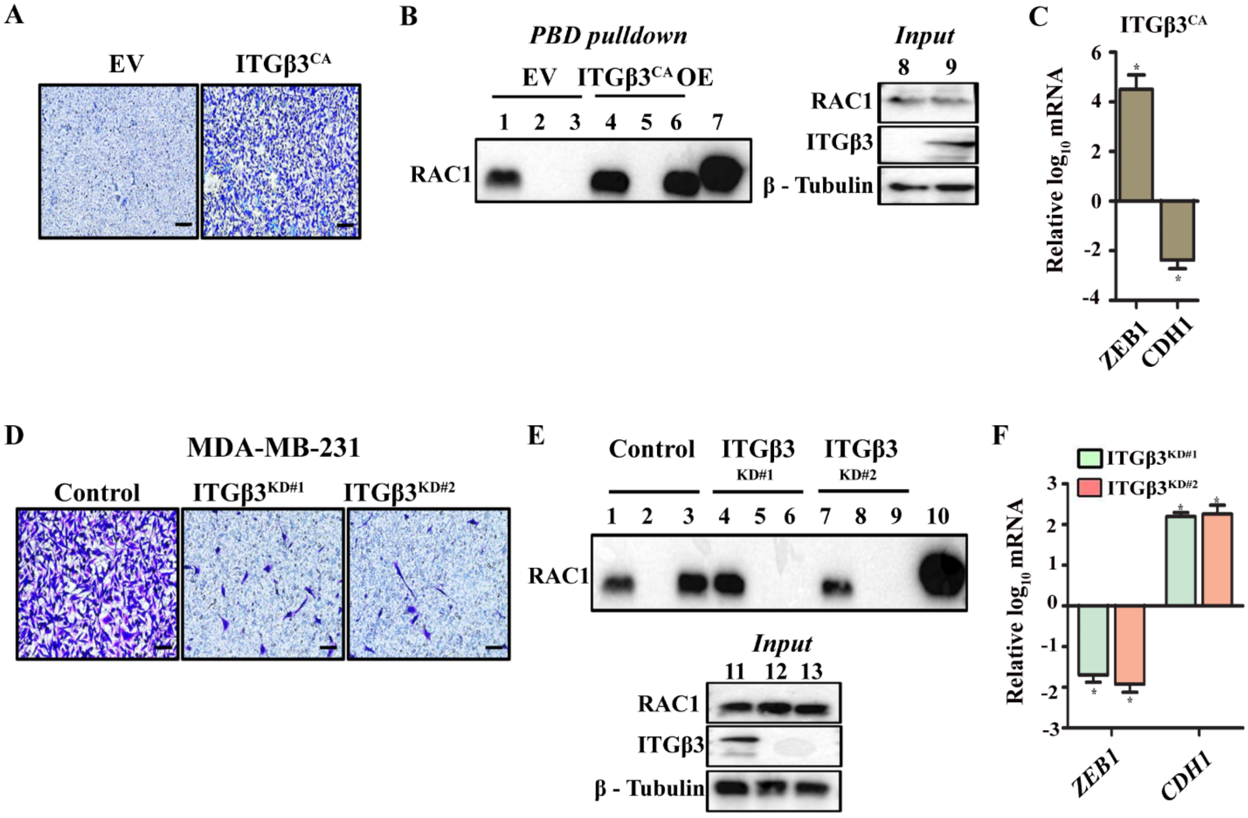
A
EV
ITGβ3CA
B
PBD pulldown
EV
ITGβ3CA OE
1 2 3 4 5 6 7
RAC1
Input
8 9
RAC1
ITGβ3
β - Tubulin
C
ITGβ3CA
Relative log10 mRNA
6
4
2
0
-2
-4
ZEB1
CDH1
D
MDA-MB-231
Control
ITGβ3KD#1
ITGβ3KD#2
E
Control
ITGβ3 KD#1
ITGβ3 KD#2
1 2 3 4 5 6 7 8 9 10
RAC1
Input
11 12 13
RAC1
ITGβ3
β - Tubulin
F
ITGβ3KD#1
ITGβ3KD#2
Relative log10 mRNA
3
2
1
0
-1
-2
-3
ZEB1
CDH1

Figure S5. Suppressing RAB4A expression in RAB4A-high cells reduces soft agar colony formation but has little impact on proliferation under the adherent culturing condition.

(A, B) Cell proliferation assay, under adherent culturing condition, on RAB4A-high PC3 (A) and SNB19 (B) cells; the comparison was made between cells with and without RAB4A knockdown. Proliferation was recorded continuously for 120 hours using IncuCyte live-cell imaging method.

(C, D) Soft agar assay on RAB4A-high PC3 (C) and SNB19 (D) cells with and without RAB4A knockdown. Top: Representative microscopic images of the colonies formed after 20 days of culture in noble agar; bottom: Quantification of relative colony numbers compared to the control using OpenCFU and Prism software. “*”, $p < 0.05$.

Figure S5

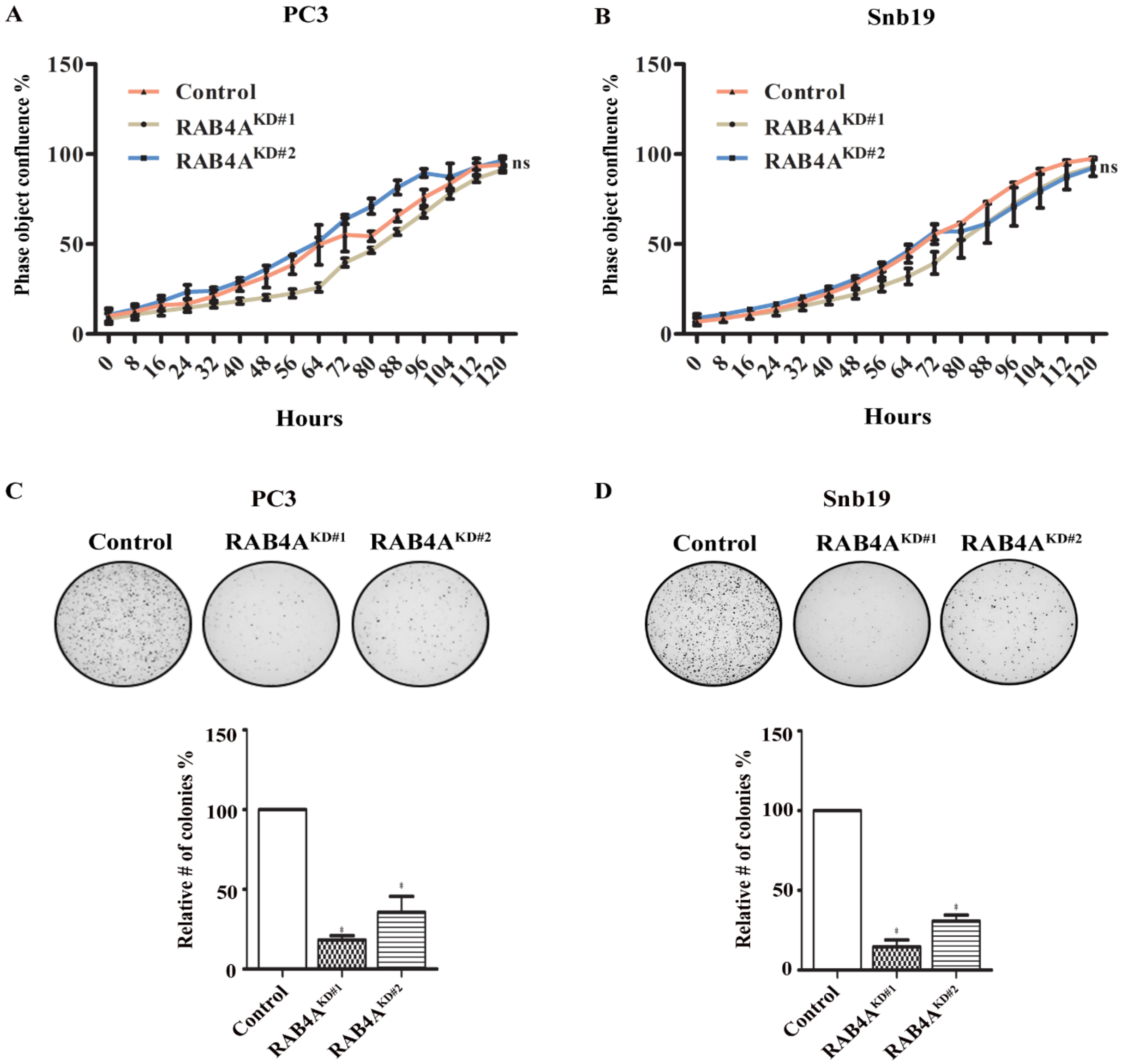
A
PC3
Phase object confluence %
150
100
50
0
Control
RAB4A^KD#1
RAB4A^KD#2
ns
0 8 16 24 32 40 48 56 64 72 80 88 96 104 112 120
Hours
B
Snb19
Phase object confluence %
150
100
50
0
Control
RAB4A^KD#1
RAB4A^KD#2
ns
0 8 16 24 32 40 48 56 64 72 80 88 96 104 112 120
Hours
C
PC3
Control
RAB4A^KD#1
RAB4A^KD#2
Relative # of colonies %
150
100
50
0
Control
RAB4A^KD#1
RAB4A^KD#2
D
Snb19
Control
RAB4A^KD#1
RAB4A^KD#2
Relative # of colonies %
150
100
50
0
Control
RAB4A^KD#1
RAB4A^KD#2

Figure S6. Wild-type RAB4A expression in RAB4A-low cells increases the soft agar colony formation ability while has little impact on adherent cell proliferation.

(A, B) The impact of RAB4A on cell proliferation, under the adherent culturing condition, on RAB4A-low MCF7 (A) and H1299 (B) cells; the comparison was made between vector control and wild-type and mutant RAB4A expressing cells. The proliferation was recorded continuously for 120 hours using IncuCyte live-cell imaging method.

(C, D) Soft agar assay on RAB4A-low MCF7 (C) and H1299 (D) cells with wild-type or mutant RAB4A protein expression as compared with vector control cells. Top: Representative microscopic images of the colonies formed after 20 days of culture in noble agar; bottom: Relative quantification of colony numbers compared to the control was analyzed using OpenCFU and Prism software. “*”, $p < 0.05$.

Figure S6

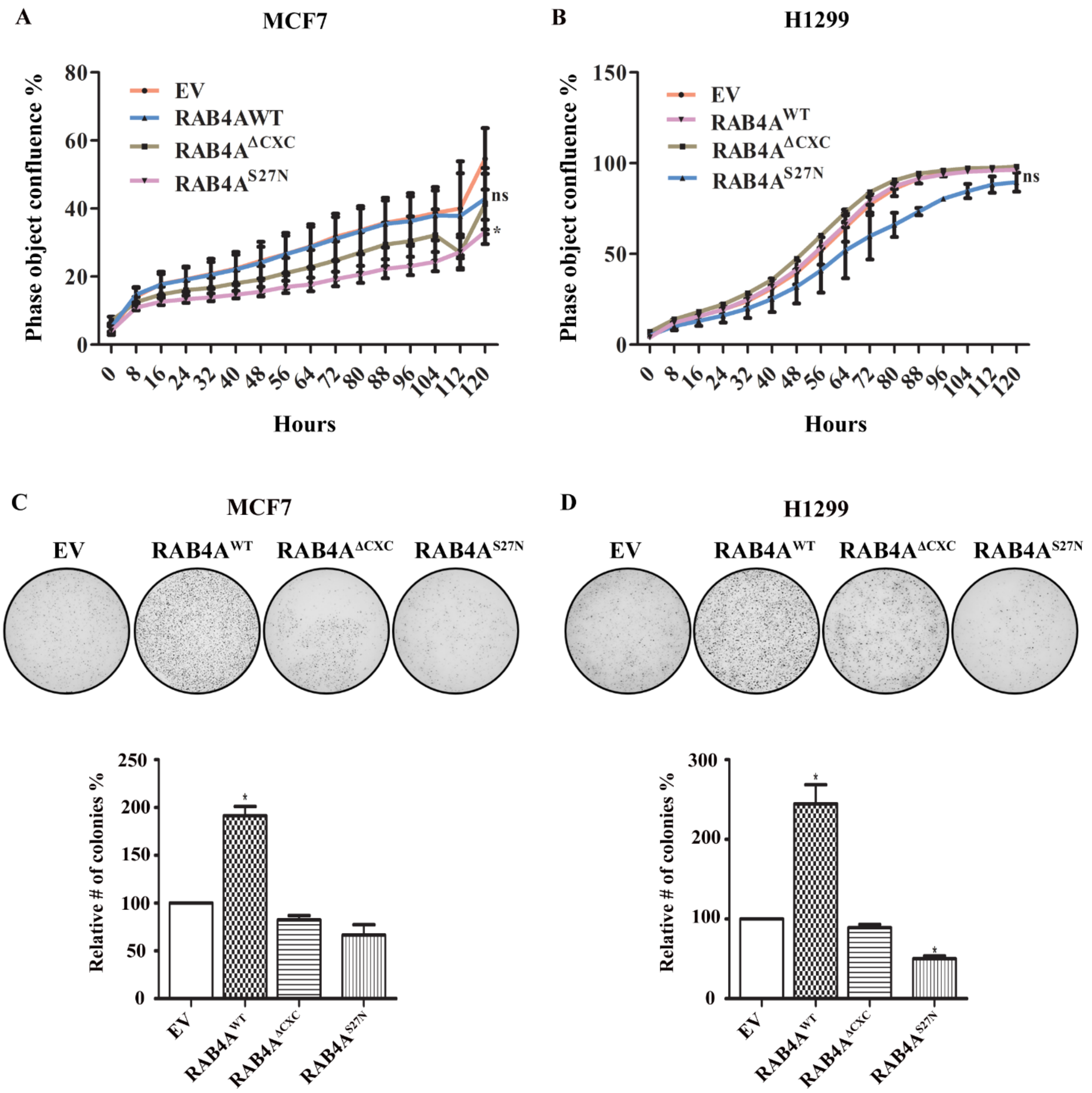

A
MCF7
Phase object confluence %
EV
RAB4AWT
RAB4A$^{\Delta CXC}$
RAB4A^{S27N}
ns
*
Hours
B
H1299
Phase object confluence %
EV
RAB4A^{WT}
RAB4A$^{\Delta CXC}$
RAB4A^{S27N}
ns
Hours
C
MCF7
EV
RAB4A^{WT}
RAB4A$^{\Delta CXC}$
RAB4A^{S27N}
Relative # of colonies %
*
D
H1299
EV
RAB4A^{WT}
RAB4A$^{\Delta CXC}$
RAB4A^{S27N}
Relative # of colonies %
*
*

Figure S7. Constitutively active, but not wild-type, RAC1 restores the MDA-MB-231 cell tumor forming ability that is lost from RAB4A knockdown in the orthotopic mammary fat pad mice model.

Representative mammary pad tumor images from each of the nine experimental groups, which are those with or without the RAB4A knockdown combined with or without the expression of RAC1. The red arrows point to the tumors after dissection. Specifically, the groups are: (1) control shRNA and empty expression vector ($Control^{KD}$ + EV), (2) control shRNA and wild-type RAC1 expression ($Control^{KD}$ + $RAC1^{WT}$), (3) control shRNA and active RAC1 expression ($Control^{KD}$ + $RAC1^{CA}$), (4) RAB4A shRNA #1 and empty expression vector ($RAB4A^{KD\#1}$ + EV), (5) RAB4A shRNA #1 and wild-type RAC1 expression ($RAB4A^{KD\#1}$ + $RAC1^{WT}$), (6) RAB4A shRNA #1 and active RAC1 expression ($RAB4A^{KD\#1}$ + $RAC1^{CA}$), (7) RAB4A shRNA #2 and empty expression vector ($RAB4A^{KD\#2}$ + EV), (8) RAB4A shRNA #2 and wild-type RAC1 expression ($RAB4A^{KD\#2}$ + $RAC1^{WT}$), and (9) RAB4A shRNA #2 and active RAC1 expression ($RAB4A^{KD\#2}$ + $RAC1^{CA}$).

Figure S7

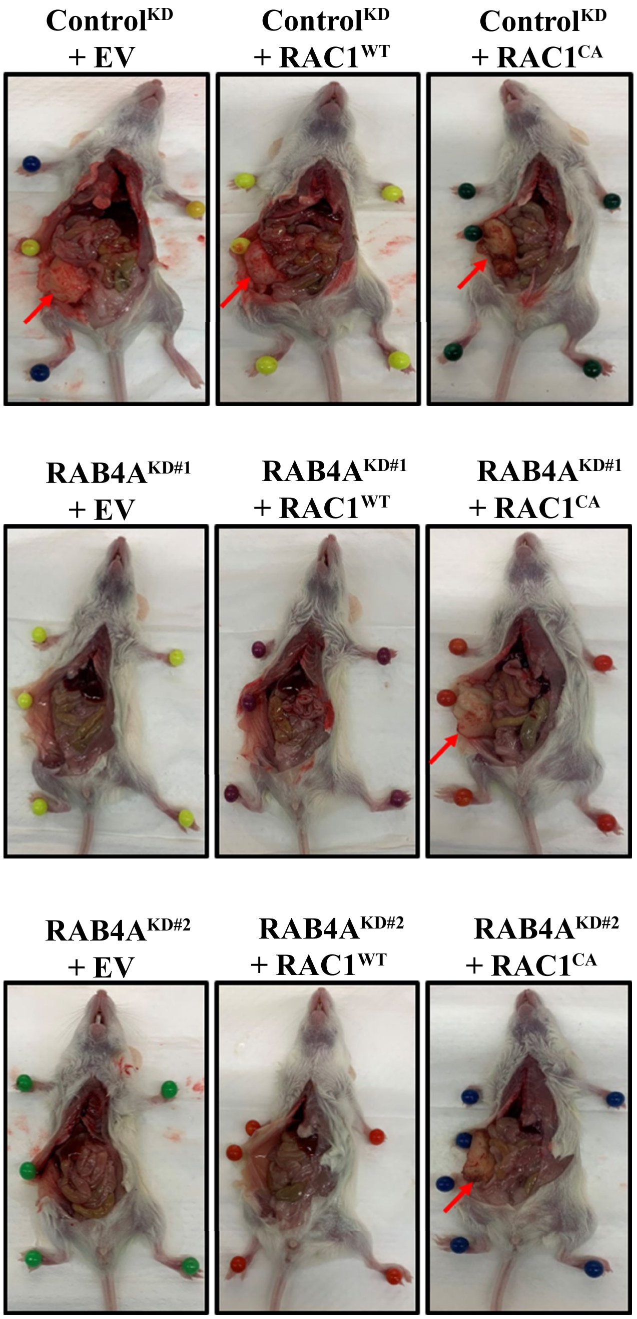
Control^{KD} + EV
Control^{KD} + RAC1^{WT}
Control^{KD} + RAC1^{CA}
RAB4A$^{KD\#1}$ + EV
RAB4A$^{KD\#1}$ + RAC1^{WT}
RAB4A$^{KD\#1}$ + RAC1^{CA}
RAB4A$^{KD\#2}$ + EV
RAB4A$^{KD\#2}$ + RAC1^{WT}
RAB4A$^{KD\#2}$ + RAC1^{CA}

References

1. Jung AR, Jung CH, Noh JK, Lee YC, Eun YG. Epithelial-mesenchymal transition gene signature is associated with prognosis and tumor microenvironment in head and neck squamous cell carcinoma. *Sci Rep.* 2020;10(1):3652.
2. Thompson JC, Hwang WT, Davis C, et al. Gene signatures of tumor inflammation and epithelial-to-mesenchymal transition (EMT) predict responses to immune checkpoint blockade in lung cancer with high accuracy. *Lung Cancer.* 2020;139:1-8.
3. Stemmler MP, Eccles RL, Brabletz S, Brabletz T. Non-redundant functions of EMT transcription factors. *Nat Cell Biol.* 2019;21(1):102-112.
